# Efficient water reduction with gallium phosphide nanowires

**DOI:** 10.1038/ncomms8824

**Published:** 2015-07-17

**Authors:** Anthony Standing, Simone Assali, Lu Gao, Marcel A. Verheijen, Dick van Dam, Yingchao Cui, Peter H. L. Notten, Jos E. M. Haverkort, Erik P. A. M. Bakkers

**Affiliations:** 1 Department of Applied Physics, Eindhoven University of Technology, 5600 MB Eindhoven, The Netherlands; 2 BioSolar Cells, P.O. Box 98, 6700 AB Wageningen, The Netherlands; 3 Department of Chemical Engineering and Chemistry, Eindhoven University of Technology, 5600 MB Eindhoven, The Netherlands; 4 Philips Innovation Services Eindhoven, High Tech Campus 11, 5656AE Eindhoven, The Netherlands; 5 Forschungszentrum Jülich (IEK-9), D-52425 Jülich, Germany; 6 Kavli Institute of Nanoscience Delft, Delft University of Technology, 2628 CJ Delft, The Netherlands

## Abstract

Photoelectrochemical hydrogen production from solar energy and water offers a clean and sustainable fuel option for the future. Planar III/V material systems have shown the highest efficiencies, but are expensive. By moving to the nanowire regime the demand on material quantity is reduced, and new materials can be uncovered, such as wurtzite gallium phosphide, featuring a direct bandgap. This is one of the few materials combining large solar light absorption and (close to) ideal band-edge positions for full water splitting. Here we report the photoelectrochemical reduction of water, on a *p*-type wurtzite gallium phosphide nanowire photocathode. By modifying geometry to reduce electrical resistance and enhance optical absorption, and modifying the surface with a multistep platinum deposition, high current densities and open circuit potentials were achieved. Our results demonstrate the capabilities of this material, even when used in such low quantities, as in nanowires.

## Abstract (web summary)

Photoelectrochemical hydrogen production from solar energy and water is one possible sustainable fuel option. Here, the authors fabricate wurtzite gallium phosphide nanowires, with a direct bandgap, allowing for enhanced optical absorption; demonstrating an enhancement in the water reduction efficiency.

## Graphical abstract

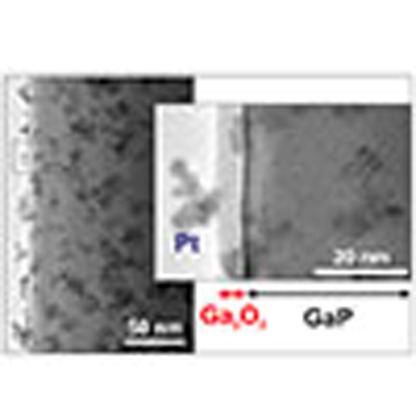

Semiconductor photoelectrochemical (PEC) cells present a promising option for direct conversion of solar energy to fuels^1^,^2^,^3^. To reach a high efficiency, it is important that the semiconductor has an optimum bandgap energy^4^ (a bandgap of 1.7–2.2 eV is required^2^), and band-edge position with respect to the redox system^5^,^6^. In addition, the semiconductor should have a good chemical stability^7^ to ensure long-term operation. Most semiconductors are limited to one side of the reaction^8^,^9^ (Fig. 1a), due to their electronic band structure, or require an external bias to achieve water splitting^10^,^11^,^12^,^13^. Materials such as Fe_2_O_3_ and TiO_2_ appear promising, and have been studied in great detail, as their bandgaps straddle the redox potentials of water (as shown in Fig. 1a). Unfortunately, the large bandgap limits the maximum solar light absorption^14^,^15^. Smaller bandgap semiconductors like Si^13^,^16^ and InP^17^, show larger solar light absorption, resulting in higher currents for nanowire arrays combined with platinum or ruthenium catalysts, but the reported *V*_OC_ for water reduction is generally too low, limiting the possibilities for full water splitting with a single junction cell.

There are, however, few semiconductor materials that come close to overcoming these limitations, such as cobalt oxide (CoO), copper oxide (Cu_2_O) and gallium phosphide (GaP). CoO nanoparticles have already demonstrated unbiased solar water splitting^18^; however, the large bandgap (2.6 eV) will limit the light absorption and, due to the nanoparticle form, gas separation will become an issue. The long-term stability of Cu_2_O in aqueous solutions is an issue, as it is difficult to control the stoichiometry, and even with precious metal catalysts the highest reported output currents are limited to ∼50% of the theoretical maximum^19^, and perhaps more importantly, the oxidation reaction has not been reported on Cu_2_O. B GaP, which conventionally has the zincblende (ZB) crystal structure, on the other hand has reasonable stability in aqueous solution^20^, can be grown with high stoichiometric control, and zinc blende (ZB) *p*-GaP photocathodes^20^,^21^,^22^ and *n*-GaP photoanodes^23^,^24^ have been demonstrated in the past. A recently reported heterojunction of *p*-GaP and *n*-TiO_2_ reported a record high *V*_OC_ of 0.71 V (versus reversible hydrogen electrode (RHE)) for ZB GaP^21^, and an *I*_SC_ of 1.5 mA cm^−2^ under AM1.5 (100 mW cm^−2^) illumination. This *I*_SC_ value is, however, far from the theoretical maximum current of 8.9 mA cm^−2^, calculated for a semiconductor with a bandgap of 2.3 eV, illuminated by one sun (AM1.5) solar radiation. The low currents reported are a direct consequence of the indirect bandgap of ZB GaP, leading to poor light absorption.

GaP nanowires can be grown with the wurtzite (WZ) crystal structure, which features a direct bandgap (2.1 eV)^25^. The direct bandgap will decrease the absorption depth of solar photons, allowing more of the absorbed charge carriers to reach the electrolyte, therefore increasing the measured current. The slightly decreased bandgap will allow more of the solar spectrum to be absorbed resulting in an increased current output of up to the theoretical maximum of 12.5 mA cm^−2^. The bandgap of the WZ GaP nanowires is well positioned with respect to the ZB GaP substrate for the reduction reaction, as seen in Fig. 1a. The valence band offset has been calculated as only 135 meV (ref. 26), and has been measured to be less, presenting little or no barrier for hole transport between the *p*-doped WZ GaP nanowire base and the *p*-doped ZB-GaP substrate. The conduction band of the ZB is higher than that of the WZ, providing an electric field, driving any excited electrons from the substrate into the nanowires. The bandgap also remains reasonably well aligned to the water reduction and oxidation potentials (Fig. 1a), theoretically maintaining GaP's ability to catalyse the oxidation reaction, and it is expected to produce the voltage required for water splitting^27^. The nanowire geometry should also improve the PEC properties as it allows decoupling of the length scales for light absorption (axial) and charge-carrier separation (radial)^28^. Periodic nanowire arrays offer the opportunity to have almost 100% light absorption across all wavelengths due to photonic effects^29^,^30^, providing light concentration in each nanowire, as well as lowering the average refractive index leading to decreased reflection losses when compared with planar samples^29^,^31^. A further possible benefit of the nanowire system is that the increased surface area decreases the current density, relaxing the requirements for electrocatalysts, meaning that earth-abundant materials should show catalytic activities similar to those of precious metals^32^. This increased surface area, however, also introduces detrimental factors. The increased junction area between the semiconductor and electrolyte will cause a decrease in photovoltage^13^,^33^, although this voltage loss is limited, as discussed further in Supplementary Note 1. There is also an increased chance of surface recombination when the surface is larger, which will decrease the concentrations of useable charge carriers^34^, and therefore also the rate of reaction.

Pure WZ *p*-GaP nanowires are grown in ordered arrays, on a *p*-GaP ZB wafer (Zn-doped (111)B GaP, AXT Inc.), for use as photocathodes for the production of hydrogen from water. We show that with optimized nanowire geometry (length, diameter) and with our newly developed catalyst deposition, we reach high PEC efficiencies, with a *V*_OC_ of >0.75 V (versus RHE) and an *I*_SC_ of >10  mA cm^−2^. The reported *V*_OC_ is higher than the current record for a ZB *p*-GaP photocathode, and is close to the flat band potential, calculated as 1.04 V (versus RHE) from Mott–Schottky plots (Supplementary Fig. 1). For a purely *p*-type photocathode with no surface modification, the flat band potential is the theoretical maximum *V*_OC_. The *I*_SC_ is higher than the theoretical maximum current for ZB GaP and close to the theoretical maximum current of 12.5 mA cm^−2^ for WZ GaP, showing the advantages of the direct bandgap nanowire system.

## Results

### WZ gallium phosphide nanowires

**Figure 1:**
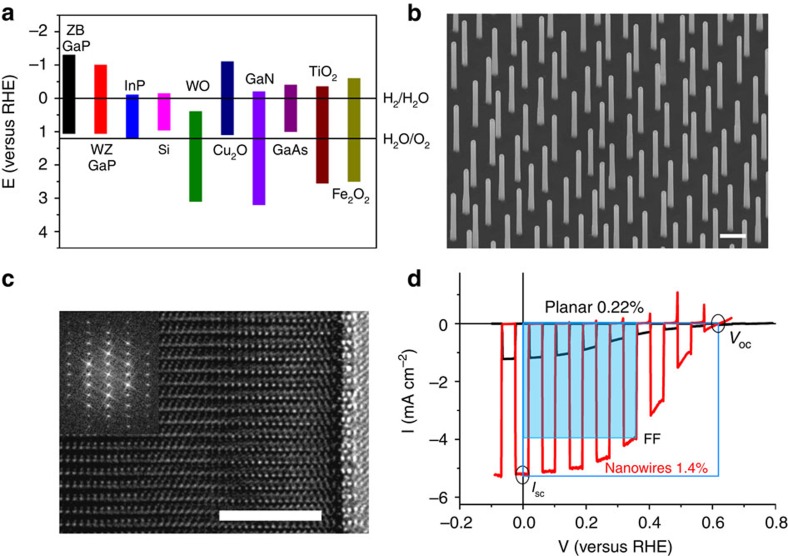
WZ *p*-type GaP nanowires for PEC water reduction. (**a**) Bandgaps and positions of several important semiconductors with respect to the reduction and oxidation potentials of water, the band-edge positions of ZB GaP and WZ GaP are calculated from Mott–Schottky plots (shown in Supplementary Information Fig. 1). (**b**) Scanning electron microscopy image of a typical array of GaP nanowires defined by nano imprint lithography. Scale bar, 400 nm. (**c**) High-resolution TEM image of a typical *p*-type GaP nanowire with WZ crystal structure; scale bar, 5 nm. The inset shows the Fast Fourier Transform of the same area. (**d**) Linear sweep voltammograms for direct comparison of nanowire (red) and planar (black) samples with molybdenum sulphide catalyst, performed under chopped 100 mW cm^−2^ AM1.5 illumination, in aqueous solution pH 0 with HClO_4_ as supporting electrolyte. Also showing open circuit potential (*V*_OC_), short circuit current (*I*_SC_) and fill factor *ff* (filled square/empty square).

Figure 1b shows a scanning electron microscopy image of a typical nanowire array. The WZ GaP nanowires are grown from a nanoimprint-patterned array of gold particles; this gives an ordered array of nanowires with 495 nm pitch and 90 nm diameter. The wires are grown with optimized parameters for the WZ crystal structure (see Methods section for more details). Figure 1c shows a high-resolution transmission electron microscopy (TEM) image of an as-grown *p*-GaP wire. The GaP wires have an almost perfect WZ crystal structure with a very low stacking fault density of <1 μm^−1^. Figure 1d compares the current density–voltage (*I*–*V*) behaviour of ZB planar (100)-oriented *p*-GaP single-crystalline substrate and WZ nanowire *p*-GaP electrodes. The nanowires used in this experiment are of optimized geometry, with lengths and diameters of ∼2.0 μm and ∼150 nm, respectively. The nanowire length is controlled by adjusting the growth time of the core. The diameter is adjusted by the growth of a shell on the nanowire surface (see Methods section for more details), as this growth method enables the shell to maintain the WZ crystal structure forming single-crystal nanowires. The planar ZB GaP surface is not insulated during experiments; however, it is expected from absorption measurements performed on nanowires, after transfer onto a poly-dimethyl-siloxane (PDMS) film (Supplementary Figs 3 and 4), that <15% of the current is due to the substrate. An amorphous molybdenum sulfide (MoS_*x*_) catalyst^35^,^36^,^37^ is deposited on both planar and nanowire samples, the catalyst will enhance transfer of charges from the semiconductor to the electrolyte, and stabilize reaction intermediates, which will reduce surface recombination. The MoS_*x*_ catalyst is deposited on the samples before all following experiments (unless stated otherwise). The *V*_OC_; fill factor (*ff*); *I*_SC_; and energy conversion-efficiency (*η*%) measured for the ZB GaP planar and nanowire-array WZ p-GaP electrodes with and without the MoS_*x*_ catalyst are listed in Table 1. We note that our reference planar ZB GaP sample already shows similar *V*_OC_ and *I*_SC_ values compared with recently reported best values for planar ZB GaP^21^. The WZ GaP nanowire sample has a higher *V*_OC_, *I*_SC_ and *ff* than the planar sample, resulting in a much higher efficiency of 1.4%. This is due to the direct bandgap of WZ GaP decreasing the absorption depth; and the nanowire geometry, decreasing reflection and bulk recombination losses^29^.

In the following sections, the steps involved in the optimization of the GaP nanowires will be discussed. This includes the study of nanowire geometry, an electrochemically produced passivation layer and a new scheme for platinum catalyst deposition.

### Nanowire geometry

**Figure 2:**
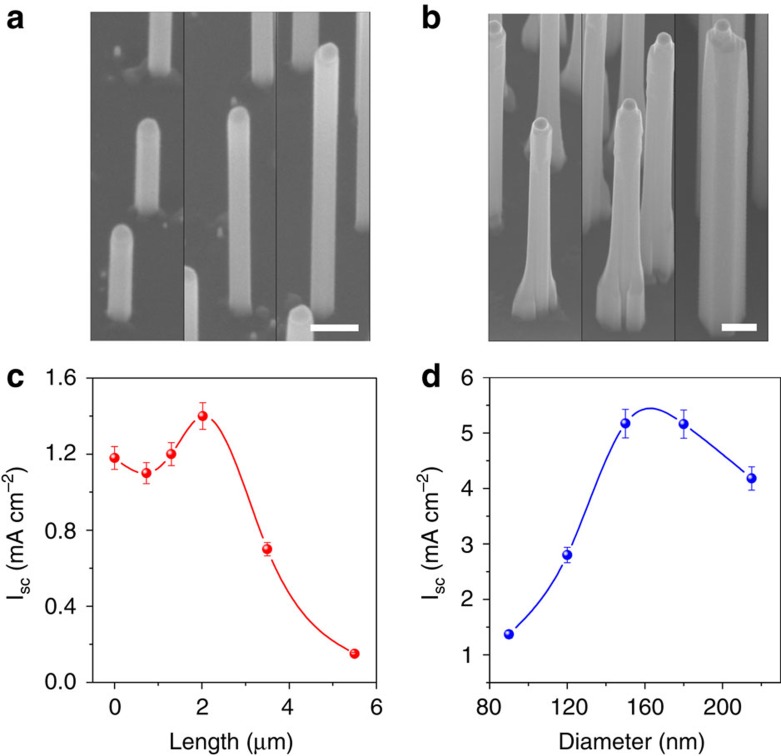
Optimization of the nanowire geometry. (**a**) Scanning electron microscopy (SEM) images of WZ GaP nanowires grown for 6, 14 and 22 min with lengths of 0.73, 1.65 and 2.16 μm, respectively. Scale bar, 200 nm for all images. (**b**) SEM images of zinc-doped WZ GaP nanowires grown for 16 min (2 μm), with an additional shell grown for 5, 10 and 30 min with diameters of 120, 150 and 215 nm, respectively. Scale bar, 200 nm for all images. (**c**) The trend observed in the short circuit current (*I*_SC_) when length is changed. (**d**) The trend observed in the *I*_SC_ when diameter is changed. Data were collected by linear sweep voltammetry, performed under chopped 100 mW cm^−2^ AM1.5 illumination, in aqueous solution pH 0 with HClO_4_ as supporting electrolyte. The error bars were calculated as two s.d. away from the average value taken from three or more experiments carried out on separate samples with the same specifications. (The data point for length 0 is for a planar (111) *p*-GaP ZB substrate).

In Fig. 2, we show that the nanowire geometry is strongly influencing the attainable *I*_SC_ in a PEC cell. We independently varied both the nanowire length and the nanowire diameter by switching between vapour–liquid–solid growth, which mainly increases the nanowire length and vapour–solid growth, which mainly increases the nanowire diameter (Fig. 2a,b). A larger nanowire length increases the solar light absorption, but if length increases too far it becomes detrimental due to an increased series resistance, which we independently measured by impedance measurements as is shown in Supplementary Fig. 2. The effect of an increased series resistance is a decrease in both *V*_OC_ and *I*_SC_. As is shown in Supplementary Fig. 3, the solar light absorption in our WZ GaP nanowires saturates below a 2-μm length, due to the direct bandgap decreasing the absorption depth, resulting in an optimum nanowire length of 2 μm. The optimum nanowire diameter is found to be 150 nm, which is determined by a trade-off between a decreasing series resistance, an increased solar light absorption and an increasing reflection loss (because of an increasing average refractive index of the layer) as nanowire diameter increases. In addition, we expect that when the nanowire diameter starts to exceed twice the space charge region thickness, calculated as 30 nm at 0 V (versus RHE) in Supplementary Fig. 1, bulk recombination starts to decrease the overall efficiency. A more detailed study of nanowire length and diameter can be found in the Supplementary Note 1 and Supplementary Figs 2 and 4.

### Molybdenum sulfide catalyst

**Figure 3:**
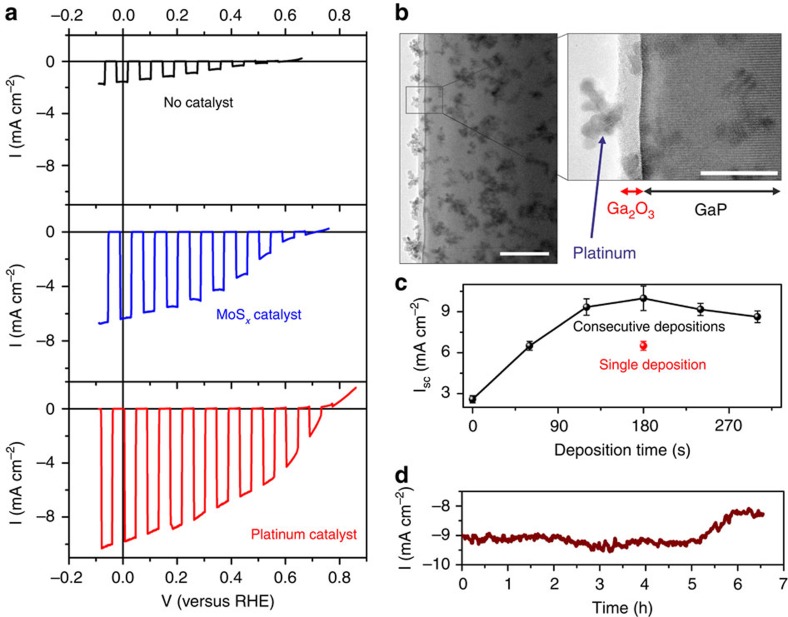
Platinum catalyst deposition. (**a**) Linear sweep voltammograms of nanowire samples with no catalyst (black), Molybdenum sulfide deposited photochemically (blue) and platinum deposited photochemically (red), (**b**) TEM images of a section of a single nanowire, of optimized geometry, after platinum has been deposited PEC for 3 × 60 s; scale bar, 50 nm, and a zoomed-in image of the same wire, with the Platinum particles, Gallium oxide and GaP nanowire clearly labelled; scale bar, 20 nm. (**c**) The trend of the short circuit current when consecutive platinum depositions are performed on the same nanowire sample (black points) and when a single long deposition is performed on a nanowire sample (red point). The error bars were calculated as two s.d. away from the average value taken from three or more experiments carried out on separate samples with the same specifications. (**d**) Long-term chronoamperometric measurement performed on nanowires after platinum catalyst deposition performed under 100 mW cm^−2^ AM1.5 illumination, in aqueous solution pH 0 with HClO_4_ as supporting electrolyte.

To fully realize the potential of GaP for water reduction, a suitable catalyst is required to promote charge transfer, thereby suppressing charge-carrier recombination. As previously mentioned, nanowires have a large surface area, and therefore a low current density, meaning that an earth-abundant catalyst such as MoS_*x*_ (refs 35, 36, 37, 38) should yield promising results. Figure 3a shows the *I*–*V* characteristics for nanowires without catalyst (black line, top panel), with MoS_*x*_ as catalyst (blue line, middle panel) and with platinum as catalyst (red line, bottom panel). Before this MoS_*x*_ deposition, an electrochemically produced oxide (EPO) passivation layer is formed. This EPO layer has been shown to improve the *I*_SC_ of uncatalysed nanowires from 1.5 to 4.1 mA cm^−2^ (see Table 1, Supplementary Note 2 and Supplementary Fig. 5). The MoS_*x*_ catalyst is deposited for an optimum deposition time of 30 s (Supplementary Fig. 6). The reaction of the precursor with the semiconductor surface also results in the formation of sulfide, which is widely known to passivate III–V semiconductors^39^,^40^,^41^. Even so, the presence of the EPO improves the overall efficiency from 1.37% (Fig. 1d) to 1.5% (Fig. 3a, middle panel), demonstrating that the EPO is a more effective passivation layer than sulfide, and will be much more important for other catalysts (that are not produced with their own passivation layer). *V*_OC_, *I*_SC_, *ff* and *η*% values for the nanowires, catalysed by MoS_*x*,_ with and without the EPO can be found in Table 1. This combination of nanowire, oxide and catalyst has already achieved the current record in *V*_OC_ of 0.71 V (versus RHE) for GaP, and has achieved a much higher *I*_SC_ (6.4 mA cm^−2^) than has yet been reported for GaP.

### Platinum catalyst

As platinum is well known to be the best catalyst for water reduction, this catalyst is implemented to explore the full potential of WZ GaP. The best performance should be achieved with a uniform particle distribution, and an average particle size of 2–5 nm (refs 42, 43). We have achieved this by a simple and cheap electroless photodeposition method (as outlined in the Methods section). Longer deposition times, as expected, result in larger platinum particles, but remarkably the number of platinum particles decreases as deposition time is increased. When the deposition time is increased from 60 to 180 s, the number of particles per 0.01 μm^2^ decreases from 100 to 34 (Supplementary Fig. 7, Supplementary Table 1 and Supplementary Note 3). This shows that the platinum deposition is a dynamic process in which larger particles are growing while smaller particles are dissolved, typical for Ostwald ripening. By interrupting the deposition process, and performing chronopotentiometry on the sample, the platinum particles are exposed to hydrogen gas, which adsorbs onto their surface^44^, changing the properties of the platinum particles, and therefore the Ostwald ripening effect during the following deposition step. Multiple deposition steps lead to a, close to optimum, particle size of 5±3 nm and a uniform particle distribution over the wire, as can be seen in Fig. 3b. There is also the added benefit in the multi-deposition case of a slightly thicker EPO layer, produced during the chronoampeometry step, improving surface passivation, as discussed in Supplementary Note 3. The trend in *I*_SC_ achieved by the interrupted deposition process is shown in Fig. 3c (black points). By performing three depositions of 60 s, a high *I*_SC_ of up to 10.9 mA cm^−2^ can be achieved. The same deposition time, without the interruptions, results in a lower *I*_SC_ of only 6.7 mA cm^−2^ (Fig. 3c red point). This is due to the poor uniformity and large particle size caused by the long continuous deposition (Supplementary Table 1). When more than three 60 s depositions are performed, the platinum particles no longer have optimum size and coverage, leading to light scattering and a decrease in current. The *I*–*V* characteristics for nanowires with the optimum platinum catalyst deposition can be seen in Fig. 3a (red line, bottom panel). With this deposition procedure, record high *I*_SC_ and *V*_OC_ values of 9.78 and 0.76 V (versus RHE), respectively, are obtained. However, the *ff* remains relatively low, below 0.4 for all samples, due to the large surface area of the nanowires. The best sample, with a *ff* of 0.39, nevertheless resulted in a record efficiency of 2.90% for a GaP large bandgap PEC cell. Higher *I*_SC_s of up to 10.9 mA cm^−2^ were recorded for other samples (Supplementary Fig. 8); however, the overall efficiency was best in the sample used for the data in Fig. 3a. The measured *I*_SC_ of >10 mA cm^−2^ corresponds to >80% of the theoretical maximum current of 12.5 mA cm^−2^. For this high efficiency WZ p-GaP device, with this level of platinum coverage, merely tens of milligrams of platinum are required for every square metre of device area. III/V devices have been shown to work well under >10 times concentrated light^2^, by combining our device geometry with light concentrators, the amount of platinum catalyst can be cut even further.

### Stability measurement

Figure 3c shows a 7 hour chronoampeometry measurement on nanowires catalysed by platinum in the presence of the EPO layer. The current starts to decreases after 5 hours, most likely due to the loss of catalyst particles as is observed by others^21^, demonstrating the promising capabilities of this system. Several gas samples were taken during this experiment, and measured by gas chromatography, giving a 97±3% Faradaic efficiency (Supplementary Fig. 9) for the hydrogen evolution reaction. This stability is not as high as is required for a commercial device, but is already higher than others have reported for unpassivated III/V PEC devices^2^,^17^ due to the conformal coverage of the EPO and catalyst particles.

## Discussion

We have found that WZ p-GaP nanowires act as an effective photocathode, due to the direct bandgap allowing for increased light absorption and the geometry allowing for good charge-carrier separation. The easy production and use of the EPO allows reasonable stabilities to be achieved. More importantly, the combination of the platinum catalyst, using the correct deposition procedure, with the WZ GaP nanowires achieves new records in *V*_OC_ and *I*_SC_, for GaP. We note here that an additional advantage of a nanowire device is that it will use only a fraction of the semiconductor material that a thin film device would use. By transferring the nanowire arrays from the growth substrate into a flexible polymer film^45^, substrate costs can be removed, and a flexible device with minimal material usage (1 g of GaP m^−2^) can be produced^46^. Nanoimprint and PDMS sample transfer are scalable technologies, which will allow for the production of large-area devices in the future. To further improve the efficiency, the doping in the core and the shell could be studied in more detail. By introducing a doping profile and an electric field, driving electrons to the nanowire surface, the *ff* should be improved^1^. Further improvement of the *ff* may also be achieved with other passivation layers, such as Al_2_O_3_ or TiO_2_ (ref. 21). These more chemically stable passivation layers should also improve the device stability^21^,^23^,^47^,^48^. We finally emphasize that direct bandgap WZ GaP is a good candidate for the wide bandgap cell in a tandem PEC device^49^,^50^.

## Methods

### Soft nanoimprint lithography

The soft PDMS stamp used here is moulded from a silicon master pattern, which contains arrays of holes fabricated by e-beam lithography. First a 300-nm poly-methyl-methacrylate (PMMA) with low element weight (35 K) is applied on cleaned 2-inch GaP (Zn-doped (111)B GaP, AXT Inc.) wafer by spin-coating and followed by a 150 °C bake. A silica sol-gel layer is then spun over the PMMA. Within 1 min after sol-gel spin-coating, the PDMS stamp is carefully applied on to the sol-gel. The structure on the PDMS stamp will be moulded to the silica sol-gel layer by capillary force. The sol-gel reacts to form silica glass in 2 h. Finally, the PDMS is removed carefully from the sol-gel layer by peeling and the pattern of an array of holes with a diameter of ∼100 nm is left. The ∼20 nm-thick silica residual between the bottom of the features and the PMMA layer is etched away by reactive ion etching with pure CHF_3_ for 50 s. Then O_2_ reactive ion etching is used to transfer the structure in the sol-gel layer into the underlying PMMA layer. The PMMA is heavily over-etched in this step to get a larger feature size in PMMA compared with sol-gel layer. After removing the oxide layer, an 8 nm-thick gold layer is deposited by evaporation. Lift off is carried out at room temperature using acetone. After spin-drying, the patterned Au particles with controlled diameter and thickness are precisely positioned.

### VLS-growth

The nanowires vapour–liquid–solid growth was performed in a low-pressure (50 mbar) Aixtron CCS-MOVPE reactor according to the procedure in ref. 22. The gold nanoparticles were patterned over the GaP (111)B substrate via nanoimprint lithography (100 nm diameter/500 nm pitch). Pre-growth annealing of the substrates under Phosphine (PH_3_) flow at 750 °C to was used to remove the oxide on the surface and the organic residuals of the lithographic process. The WZ wire growth was performed at 750 °C using tri-methyl gallium and Phosphine (PH_3_) as precursor gases at molar fractions of 7.4 × 10^−5^ and 1.7 × 10^−3^, respectively, with a total flow of 8.2 l min^−1^ using hydrogen as carrier gas. HCl flow (molar fraction 1.2 × 10^−4^) was introduced to suppress the radial overgrowth of the wires. The shell growth was performed at 700 °C using tri-methyl gallium and PH_3_ molar fractions of 5.4 × 10^−5^ and 1.1 × 10^−2^, without any HCl flow. To induce a *p*-type doping, a Di-Ethyl Zinc (DEZn) flow (molar fraction 3.4 × 10^−4^) was used during both the core and the shell growths.

The NW samples used in Fig. 2 were grown for 6–10–14–18–22 min. In the samples used in Fig. 3, the GaP core was grown for 16 min, followed by the shell growth of 5–10–20–30 min.

### Electrode fabrication

To make an ohmic back contact, layers of Ti/Au (50/100 nm) are evaporated onto the back of the *p*-GaP substrate and annealed at 250 °C for 10 min. The p-GaP samples are then cleaved into small pieces and fitted into a PEEK holder with 0.07 cm^2^ hole exposed to the electrolyte.

### Photochemical electroless MoS_*x*_ deposition

The cleaned GaP samples are immersed into a 1-mM (NH4)_2_[MoS_4_] solution, which is freshly prepared. The sample is then illuminated for 30 s by a 400-nm LED under 1.4 mW cm^−2^ intensity, the generated electrons in the conduction band of the *p*-GaP reduce [MoS_4_]^2−^ at the surface to form amorphous MoS_*x*_ and the generated holes in the valence band oxidize sulfide. Increasing the deposition time up to 5 min has little effect on the catalytic behaviour of the MoS_*x*_.

### Photochemical electroless platinum deposition

The cleaned GaP samples are immersed into a 50-mM H_2_PtCl_6_, 2 M HCl aqueous solution. The sample is then illuminated for a set time by a 400-nm LED under 1.4 mW cm^−2^ intensity, the generated electrons in the conduction band of the *p*-GaP reduce the Pt^4+^ at the surface to form platinum metal and the generated holes in the valence band oxidize the [H_2_Cl_6_]^4−^ to HCl and Cl_2_. For consecutive depositions a chronopotentiometry step of 5 min, under chopped AM1.5 1 sun illumination, was performed between depositions. Optimum performance was found for three consecutive 60 s deposition steps, with a decrease in performance seen after further depositions.

### PEC measurements

The PEC measurements are performed in a three-electrode electrochemical cell with a saturated calomel reference electrode and a Pt foil counter electrode in 1 M HClO_4_ (Sigma-Aldrich) electrolyte. The reference potentials reported here are converted to the RHE potential for convenience. The current–potential curves are measured by Autolab 302 N (Eco Chemie, Metrohm). 100 mW cm^−2^ AM 1.5G illumination is provided by a 300-W Xenon Lamp (Newport 67005) with AM 1.5 G filter (Newport 81094). The spectrum and intensity of the lamp is calibrated by a spectroradiometer (IL90 International Light).

The *I*_SC_ is defined as the photocurrent magnitude at 0 V versus RHE. The *V*_OC_ is defined as the potential where the measured current is 0 A. The *ff* is defined as the ratio of 
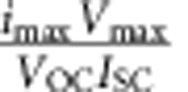
 where *i*_max_ and *V*_max_ are the photocurrent, and potential at the maximum power point of the system.

The efficiency is calculated as:


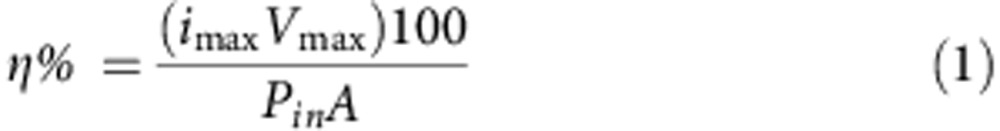


Where *P*_in_ is the intensity of the light incident on the sample (mW cm^−2^), and *A* is the sample area (cm^2^)^51^. The efficiency calculated for the photocathode is referenced to a hypothetical cathode with no overpotential losses at 0 V versus RHE^52^.

### Absorption measurements

The absorption data were derived from wavelength-dependent separate total transmittance (*T*(*λ*)) and total reflectance (*R*(*λ*)) measurements. The GaP nanowires were embedded in PDMS using the procedure described by Standing *et al*.^45^. The PDMS film with nanowires was stuck onto an aperture of a Thorlabs integrating sphere on which a silicon photodetector was mounted. An Energetiq EQ-99 lamp combined with a Princeton Instruments monochromator and chopper produced a light beam to illuminate the sample, while the lock-in amplified silicon photodetector was used to detect the light integrated in the sphere. For the transmittance measurement, the sample was mounted on the only open aperture of the sphere, to integrate all light transmitted through the sample. For the reflectance measurement, the sample was mounted on one side of the sphere under a small oblique angle (<6°, to collect also the specular reflection) and illuminated through the opposite aperture of the integrating sphere, to integrate all light reflected by the sample. Both measurements were corrected for the background and divided over a reference measurement. The references were measured without a sample mounted in the transmittance configuration, and with a piece of perfectly reflecting integrating sphere material mounted in the reflectance configuration, respectively. The wavelength range of the incident light, limited by the signal-to-noise ratio of the measurement, was set from 407 to 720 nm, with a 0.5-nm step size. The absorptance fraction *A*(*λ*) was derived by using *A*(*λ*)=1−*T*(*λ*)−*R*(*λ*). The absorbed power of the AM1.5G solar spectrum was calculated by multiplication of the absorptance fraction by this AM1.5G, and integrated over the used wavelength range.

### Characterization

Scanning electron microscopy images are obtained with a Zeiss Sigma microscope. TEM studies were performed using a JEOL JEM200 ARM probe corrected TEM, operated at 200kV.

## Additional information

**How to cite this article:** Standing, A. *et al*. Efficient water reduction with gallium phosphide nanowires. *Nat. Commun.* 6:7824 doi: 10.1038/ncomms8824 (2015).

## Supplementary Material

Supplementary Information Supplementary Figures 1-9, Supplementary Table 1, Supplementary Notes 1-3 and Supplementary References

## Figures and Tables

**Table 1:** Planar and nanowire performance.

**Sample**	***V***_**OC**_ **(V versus RHE)**	***I***_**SC**_ **(mA cm**^−2^)	***ff***	***η*****%**
Planar, no catalyst	0.677	1.18	0.21	0.17
Planar, MoS_*x*_	0.726	1.21	0.25	0.22
NW, no catalyst	0.62	1.5	0.27	0.28
NW, with EPO	0.75	4.1	0.18	0.55
NW, MoS_*x*_, no EPO	0.66	5.6	0.37	1.37
NW, MoS_*x*_ with EPO	0.71	6.4	0.33	1.50
NW, Pt 1 × 60 s, with EPO	0.73	6.5	0.18	0.85
NW, Pt 3 × 60 s, with EPO	0.76	9.8	0.39	2.90
NW, Pt 1 × 180 s, with EPO	0.73	6.7	0.20	0.98

EPO, electrochemically produced oxide; *ff*, fill factor; NW, nanowire.

A summary of the efficiency measured for different planar and nanowire samples after different catalyst depositions have been performed.
