# Supplementary material for: Efficient water reduction with gallium phosphide nanowires
Source: Nat Commun. 2015 Jul 17;6:7824. doi: 10.1038/ncomms8824 (PMC4518318; doi:10.1038/ncomms8824)
Supplement: Supplementary Information — Supplementary Figures 1-9, Supplementary Table 1, Supplementary Notes 1-3 and Supplementary References [file ncomms8824-s1.pdf]

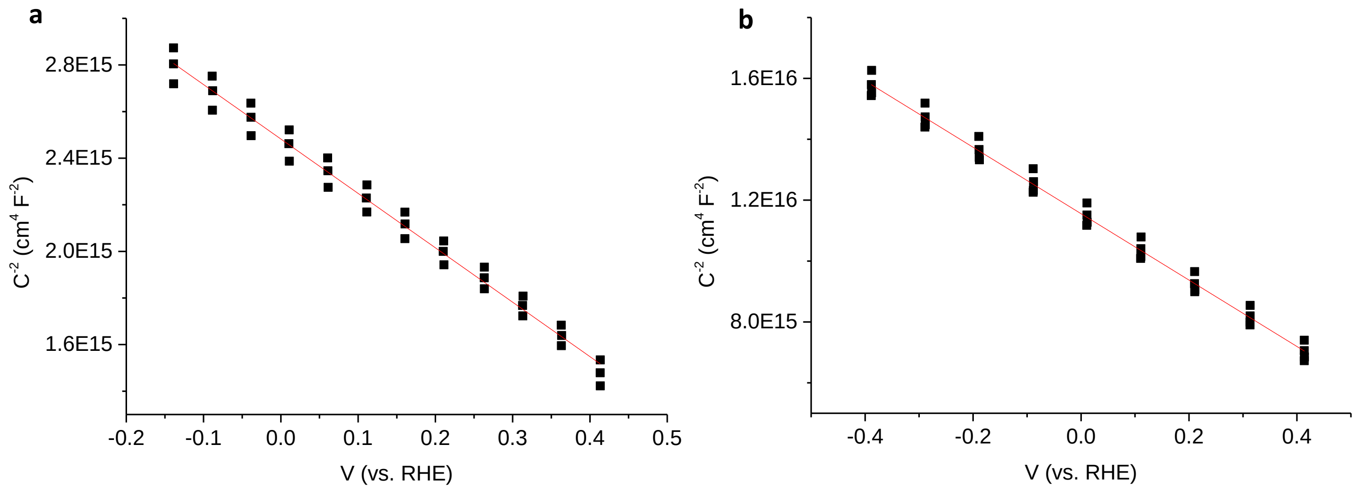

Sample	Linear fit equation	V_{FB} vs.RHE (x-Intercept)	Dopant concentration (cm^{-1})	Space charge region width at 0V (vs. RHE)
ZB GaP Planar	y = -2.34E+15x + 2.48E+15	1.06E+00	6.81E+18	14nm
WZ GaP Nanowires	y= -1.09E+16x + 1.16E+16	1.06E+00	1.46E+18	30nm

Supplementary Figure 1. Mott-Schottky plots. Impedance measurements were performed in the dark, in aqueous solution pH0 with $HClO_4$ as supporting electrolyte. Mott-Schottky plots for (a) ZB GaP planar and (b) WZ GaP nanowire samples were calculated from the Mott-Schottky equation, which can be written as;

$$\frac{1}{C_{SC}^2} = \frac{2}{q\varepsilon_0 \varepsilon N}(V - V_{FB} - \frac{kT}{q}) \quad (1)$$

where C_{SC} is the capacitance of the space charge region, q is the elementary charge, $\mathcal{E}_0$ is the permittivity of free space, $\mathcal{E}$ is the dielectric constant of ZB GaP, N is the dopant concentration, V is the applied potential, V_{FB} is the flat band potential and approximate valence band position (vs. RHE) for a p-type semiconductor, k is the Boltzmann constant and T is the temperature in Kelvin. The data from the Mott-Schottky equation allows that calculation of the space charge width, which can be written as;

$$W^2 = \left(\frac{2q(V - V_{FB})}{kT}\right)^{1/2} \quad (2)$$

where W is the space charge region width. The table shows; the flat band potential (V_{FB}), the dopant concentration, and the width of the space charge region at 0V, for the ZB planar and WZ nanowire GaP samples used.

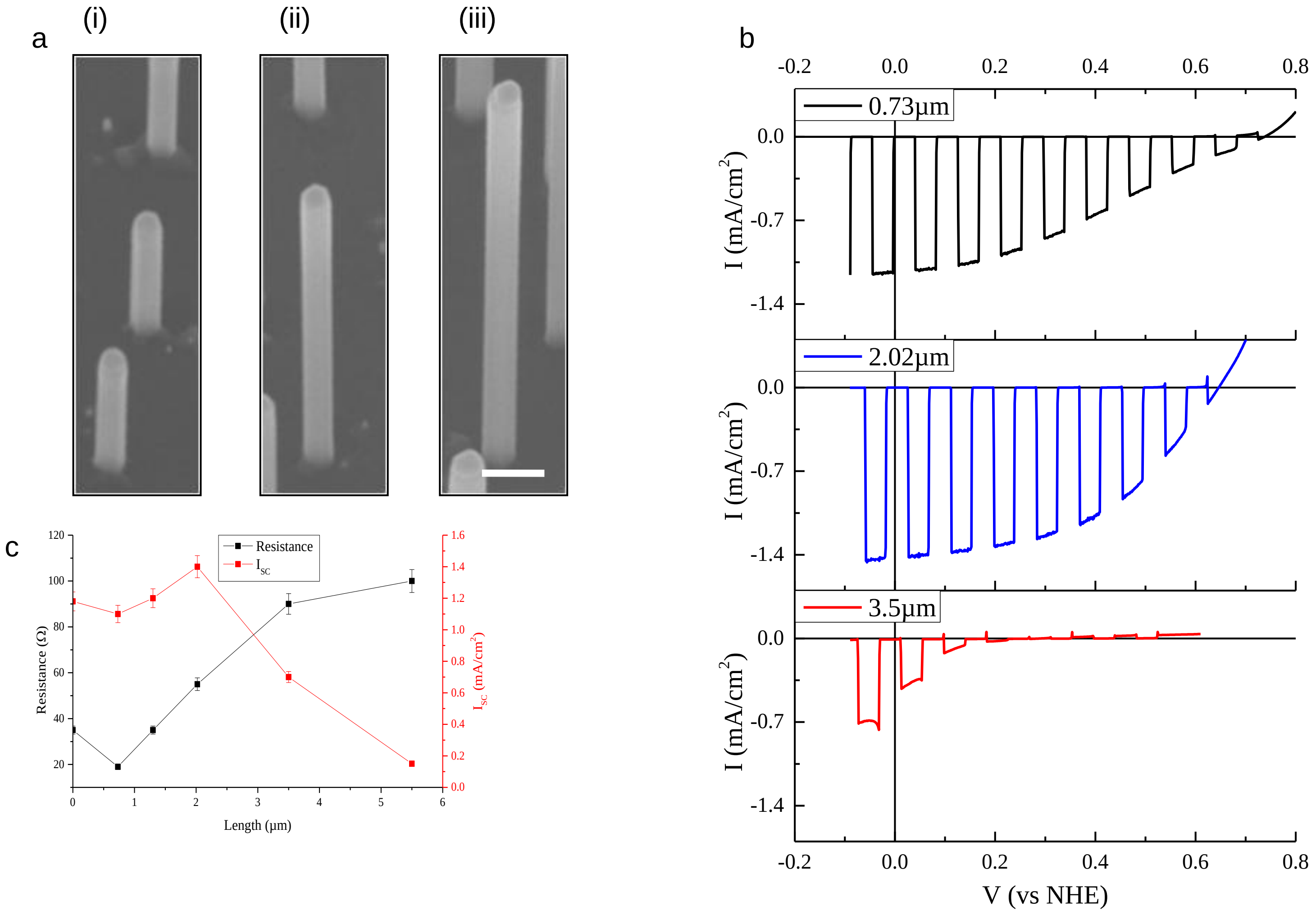

Supplementary Figure 2. The effect of nanowire length on photoelectrochemical performance. a) SEM images of Zinc-doped WZ GaP nanowires grown for (i) 6min, (ii) 14min, and (iii) 22min with lengths of 0.73μm, 1.65μm and 2.16μm respectively. Scale bar 200nm for all images. **b)** Linear sweep voltammograms of NW samples with NW lengths of 0.73μm (black/ top), 2.02μm (blue/ middle) and 3.5μm (red/ bottom), performed under chopped 100mW/cm^2 AM1.5 illumination, in aqueous solution pH0 with $HClO_4$ as supporting electrolyte. **c)** Plots of resistance (black, left y-axis) and I_{SC} (red, right y-axis) against nanowire length. The error bars were calculated as two standard deviations away from the average value taken from 3 or more experiments carried out on separate samples with the same specifications

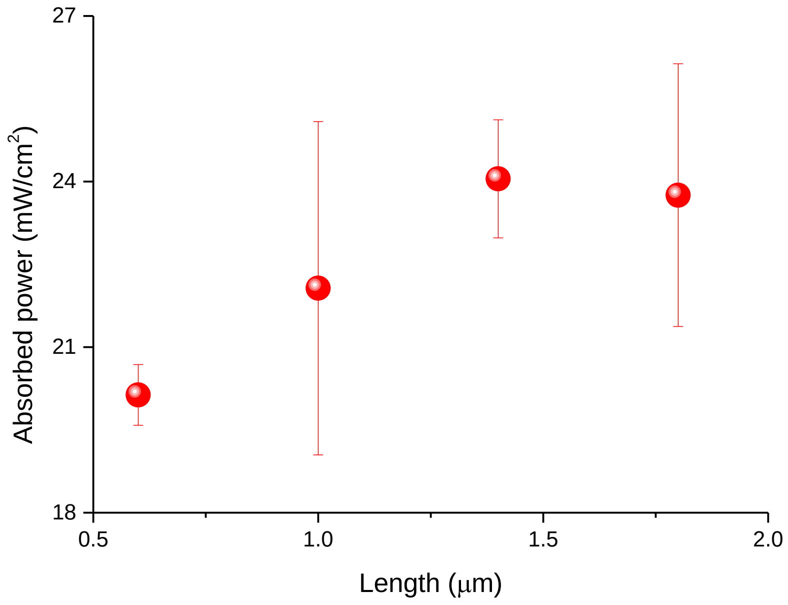

Supplementary Figure 3: The trend of light absorption with increasing wire length. The nanowire samples with different lengths are transferred into PDMS[1] and absorption measurements were carried out as outlined in section S4.7, resulting in an absorption fraction spectrum. At each wavelength, the absorption fraction was multiplied by the power density of the AM1.5G spectrum at this wavelength, in order to obtain the absorbed power density at this particular wavelength, if excited with AM1.5G. Then, this power density was integrated over the measured wavelength range, to obtain the total absorbed power per unit area. The measurements were repeated for several different positions on the nanowire samples. The error bars indicate the spread of the calculated powers for each nanowire length.

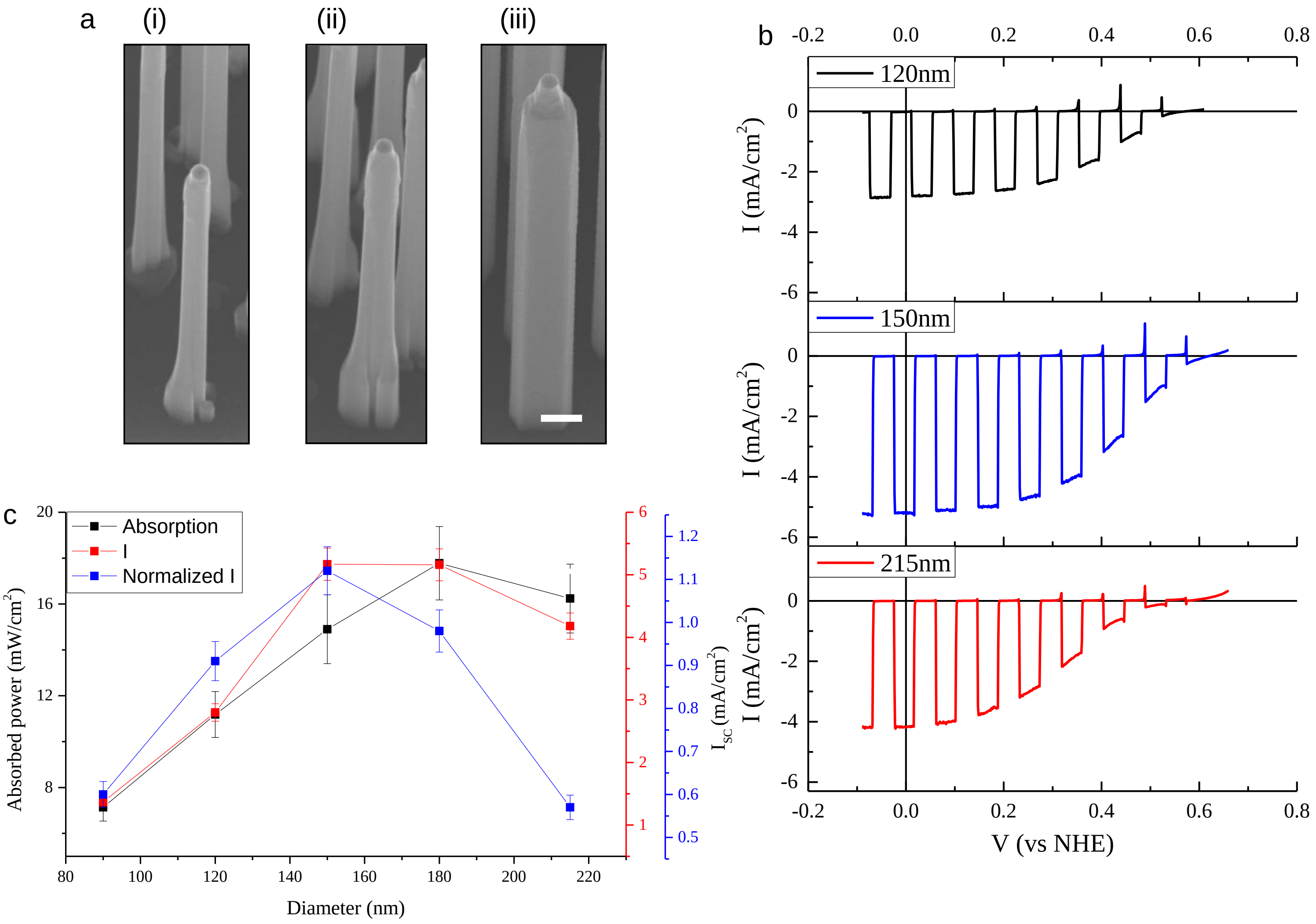

Supplementary Figure 4. The effect of nanowire diameter and surface area on photoelectrochemical performance. a) SEM images of the p-type WZ GaP nanowires grown for 16min (2µm), with additional shell grown for (i) 9min, (ii) 10min and (iii) 30min with respective diameters of 120nm, 150nm and 215nm. Scale bar 200nm for all images. **b)** Linear sweep voltammograms of NW samples with NW diameters of 120nm (black/top), 150nm (blue/middle) and 215nm (red/ bottom), performed under chopped 100mW/cm^2 AM1.5 illumination, in aqueous 1M $HClO_4$ solution. **c)** Plots of light absorption, measured as defined in figure S3 (black, left y-axis), I_{SC} (red, 1st right y-axis) and I_{SC} normalized to the nanowire sidewall and substrate surface area (blue, 2nd right y-axis) against nanowire diameter. The error bars were calculated as two standard deviations away from the average value taken from 3 or more experiments carried out on separate samples with the same specifications

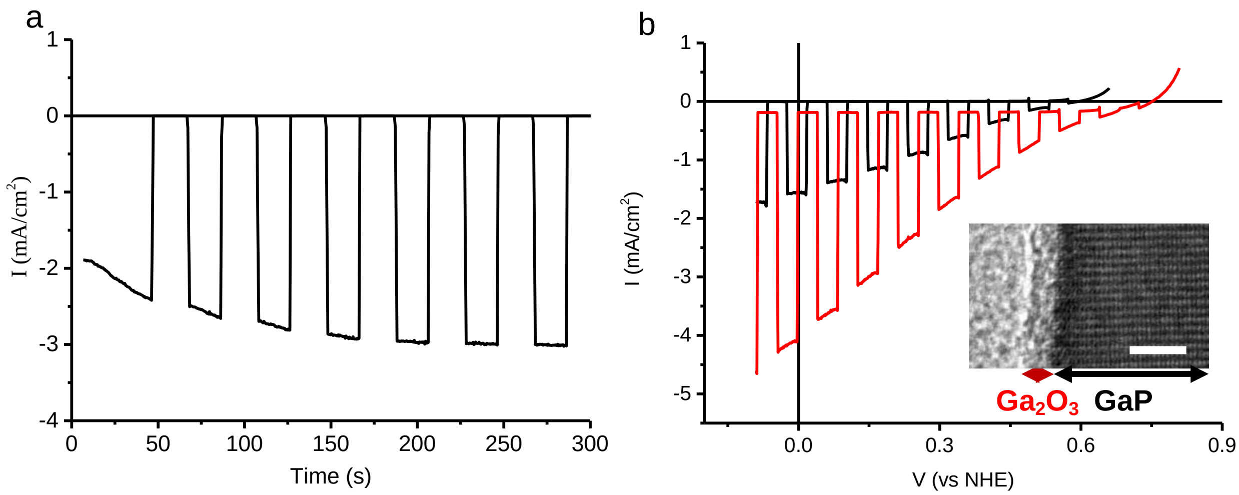

Supplementary Figure 5: The Electrochemically Produced Oxide. a) Chronopotentiometry measurement of a WZ GaP nanowire sample without catalyst, showing the production of the EPO. Experiment is performed at 0V (vs. RHE) under chopped 100mW/cm^2 AM1.5 illumination, in aqueous solution pH0 with $HClO_4$ as supporting electrolyte. b) Linear sweep voltammogram of a WZ GaP nanowire sample before (black) and after (red) EPO deposition, performed under chopped 100W/cm^2 AM1.5 illumination, in aqueous solution pH0 with $HClO_4$ as supporting electrolyte. The inset is a TEM image of a section of a single nanowire, of optimized geometry, after EPO deposition scale bar is 5nm. Different samples were used for figures a and b.

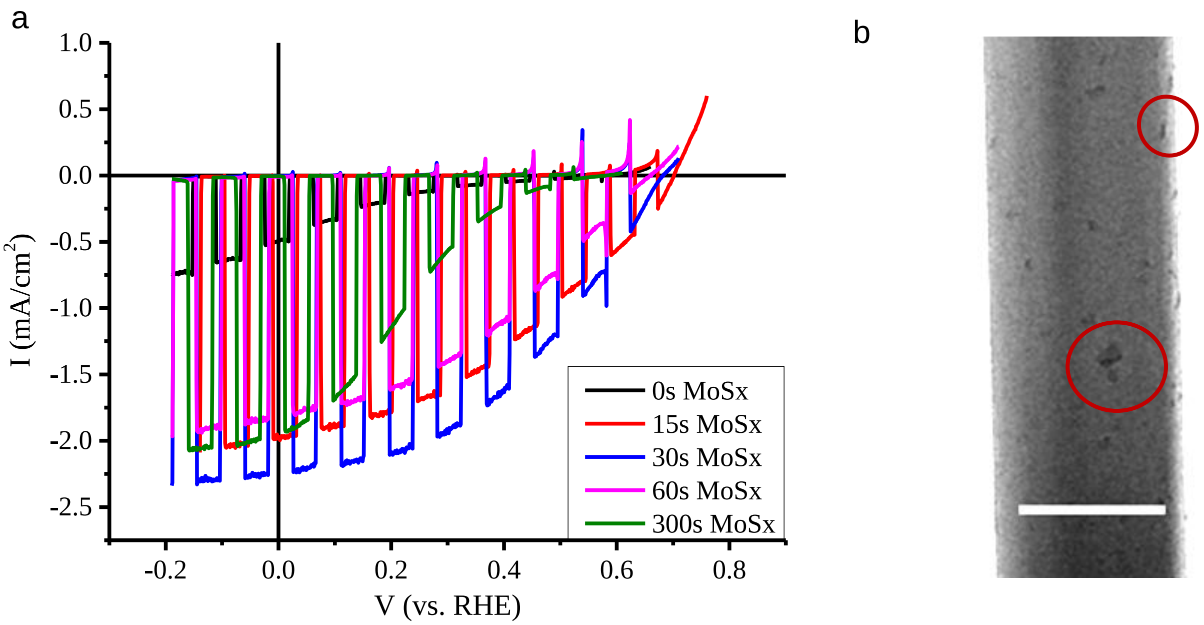

Supplementary Figure 6: MoSx Optimization a) Linear sweep voltammograms of 120nm diameter nanowire samples with MoSx deposited photochemically for 0s (black line), 15s (red line), 30s (blue line), 60s (pink line) and 5minutes (green line) performed under chopped 100W/cm^2 AM1.5 illumination, in aqueous solution pH0 with $HClO_4$ as supporting electrolyte. **b)** High resolution TEM image of a nanowire after MoS_X has been deposited for 30s, red ovals are used to highlight the position of some MoS_X particles. Scale bar is 100nm.

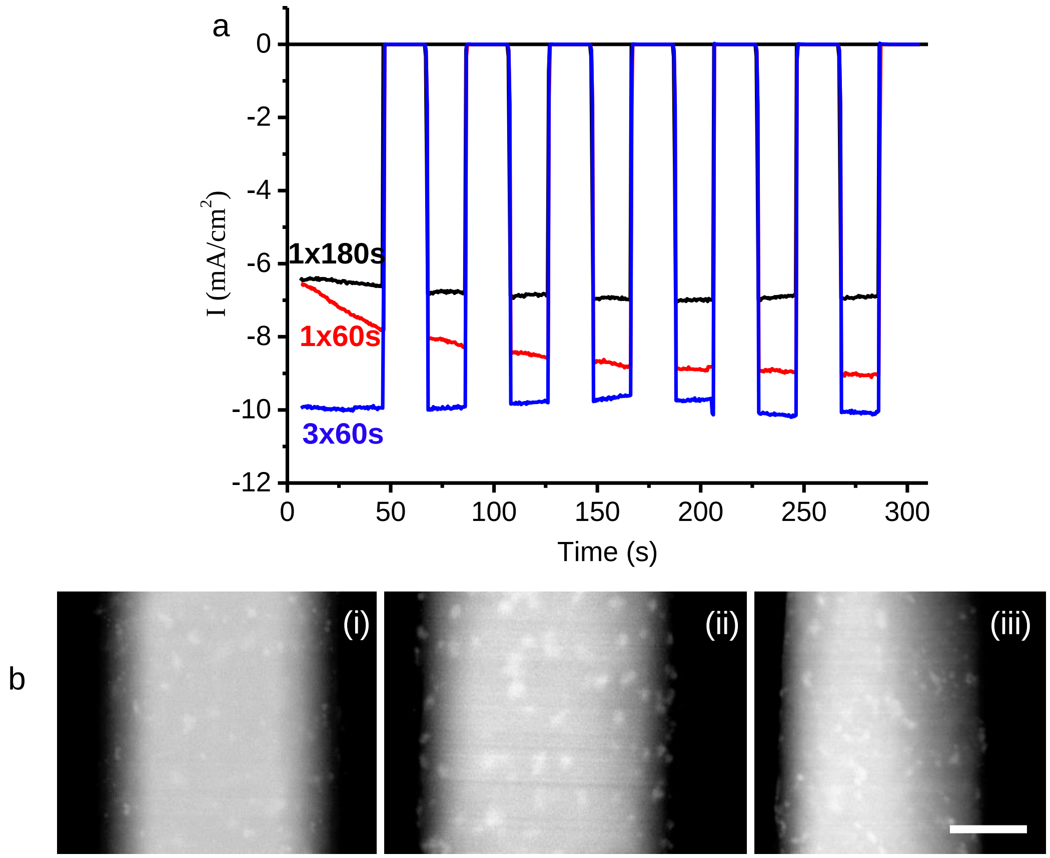

Supplementary Figure 7: Platinum Catalyst a**)** Chronopotentiometry of NW samples after Platinum has been deposited photochemically for 1x180s (black line) 1x60s (red line) and 3x60s (blue line). Performed at 0V (vs. RHE) under chopped 100mW/cm^2 AM1.5 illumination, in aqueous solution pH0 with $HClO_4$ as supporting electrolyte. b**)** Dark field TEM images of single nanowires after platinum has been deposited photoelectrochemically for 1x60s (i), 3x60s (ii) and 1x180s (iii), scale bar is 50nm.

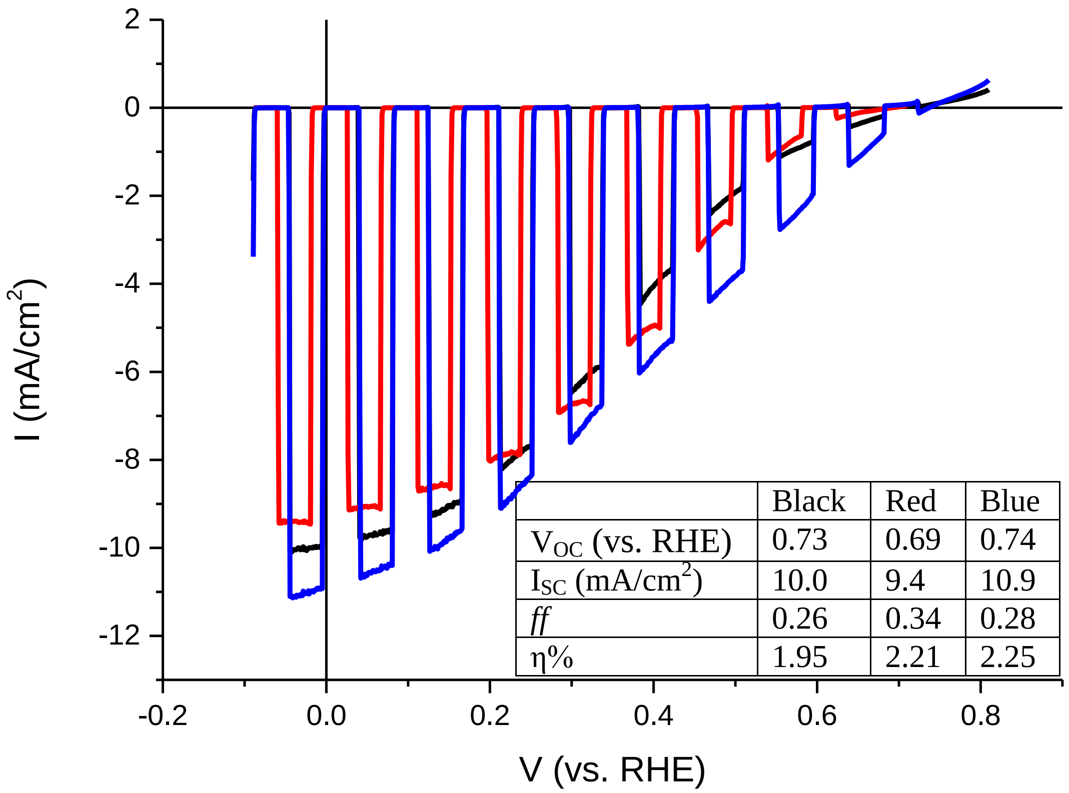

	Black	Red	Blue
V_{OC} (vs. RHE)	0.73	0.69	0.74
I_{SC} (mA/cm^2)	10.0	9.4	10.9
ff	0.26	0.34	0.28
η%	1.95	2.21	2.25

Supplementary Figure 8: 3x60s Pt repeats Linear sweep voltammograms of three identical 120nm diameter nanowire samples with Pt deposited photochemically for 3x60s performed under chopped 100W/cm^2 AM1.5 illumination, in aqueous solution pH0 with $HClO_4$ as supporting electrolyte. The inset table shows the V_{OC}, I_{SC}, *ff* and η% values taken from each curve.

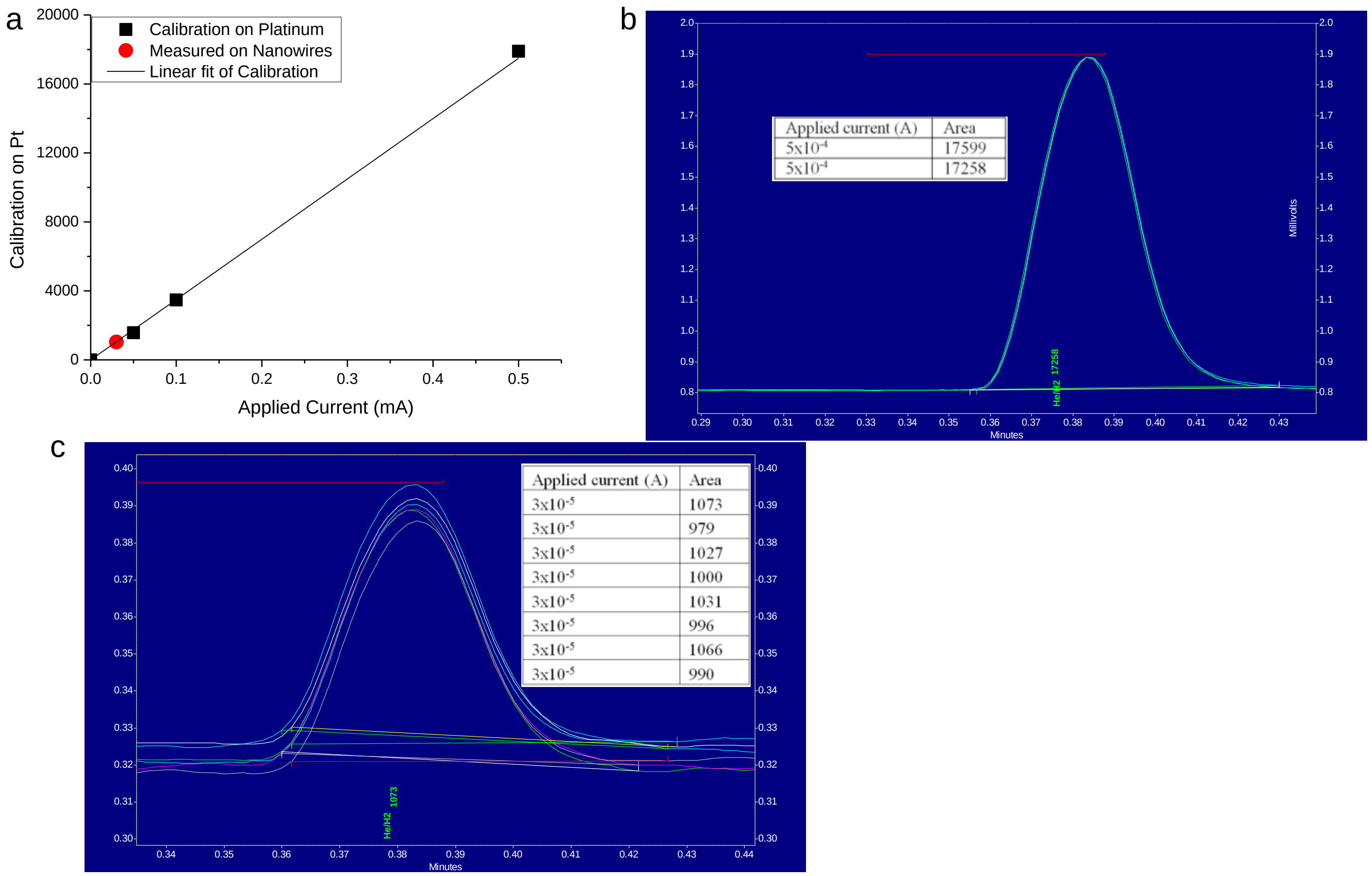

Supplementary Figure 9: Gas Chromatography a) Measurements were taken from a platinum electrode with different applied currents of 0mA, 0.05mA, 0.1mA and 0.5mA to form the calibration line (black points). The calibration line has an R^2 value of 0.992, with small errors of ±2% for each measured value. The red point is from a measurement carried out on a nanowire sample after a 3x60s Pt deposition. b) The data taken from the gas chromatography (GC) measurement carried out on the platinum electrode with an applied current of 0.5mA. c) The data taken from the GC measurement carried out on the nanowire electrode. Several samples were taken over a four-hour period during the long time chronopotentiometry measurement shown in the main text figure 3.c, a potential of 0V (vs. RHE) was applied, and the current was measured to be $3x10^{-5}$A due to the small size of the sample, the measured data can be seen in the inset table. The Faradaic efficiency calculated from this measurement is 97.6% with an error (calculated as 1 standard deviation from the average) is found to be ±3%.

Supplementary Table 1: Platinum catalyst depositions: A summary of the platinum particle size and distribution after different types of deposition.

**Deposition Process**	**Particle size (nm)**	**Particles per 100nmx100nm square of nanowire surface**
1x60s	3.5±2.5	100±10
3x60s	5±3	50 ±6
1x180s	14.5±12.5	34±16

Supplementary note 1. Geometry optimization

1.1 Wire Length:

In the following sections we will discuss the geometry optimization, and catalyst deposition steps studied to reach high efficiency. We first study the effect of **wire length**. Supplementary figure 2.a shows SEM images of wires with different lengths without catalysts. In supplementary figure 2.b the I-V behavior, of wires with lengths 0.73µm, 2.02µm and 3.5µm and a constant diameter of 90nm, is shown. The shortest wires (0.73µm, black line/ top panel) exhibit the highest V_{OC}, however, when the length is increased to 2.02µm (blue line/ middle panel), the I_{SC} and *ff* improve, with only a small decrease in V_{OC}. With a further increase in length to 3.5µm (red line/ bottom panel) a dramatic decrease is observed in V_{OC}, I_{SC} and *ff*, however the saturation current appears unchanged. In supplementary figure 2.c the measured series resistance in the system, obtained from impedance measurements, in the dark (black points, left axis), and the I_{SC}, under illumination (red points, right axis), are plotted. The lines connecting the points in supplementary figure 2.c are only added as a guide to the eye. In supplementary figure 2.c (black points) we see that resistance increases greatly as nanowire length increases, as is expected from the equation;

$$R = \rho L / A \qquad (3)$$

where R is the resistance of the wire, ρ is the resistivity of the material, L is the wire length, and A is the wire cross sectional area. However this is clearly not the only factor affecting performance as the trend for I_{SC} is not the inverse of resistance. This is due to the light absorption increasing with nanowire length (supplementary figure 3). Due to the increased surface area, the flux of electrons through the electrode electrolyte junction per unit area decreases. This causes a decrease in the quasi-Fermi level, and therefore V_{OC}[2–4]. The change in V_{OC} can be calculated by

$$V_{OC} = \frac{k_B T}{q} \ln\left(\frac{I_{SC}}{\gamma I_0}\right) \tag{4}$$

where γ is the actual junction area and I_0 is the saturation current density. This factor, however, only accounts for a small decrease in V_{OC} on the order of 10s of mV, the further drop in voltage is due to the resistance and length of the nanowire. As the nanowire length continues to increase, more voltage is lost due to the increased resistance and surface area, causing the decrease in I_{SC} observed in supplementary figure 2.c, and the change in the I-V curve shape observed in supplementary figure 2.b. It is found that the optimum wire length is 2µm, yielding promising V_{OC} and *ff*s; this wire length allows for good transport of charge carriers and reasonable absorption of light without too much voltage drop from the increased resistance and surface area. The $I_{SC,}$ however, should be able to reach a much larger value of up to the previously mentioned value of; 12.5mA/cm^2.

1.2 Wire diameter:

Using the optimized wire length, the effect of the **wire diameter** is studied by growing a shell on the wires, maintaining the pure WZ crystal structure, with nominally the same dopant concentration as used for the growth of the core. Average wire diameters of 90nm, 120nm, 150nm, 180nm and 215nm are obtained by respective shell growth times of 0, 5, 10, 20 and 30 minutes. Supplementary figure 4.a shows the SEM images of the wires with 5, 10, and 30 minute shell growth times. It is evident from these SEM images that the wire length also increases with shell growth; this is due to unavoidable axial growth through the catalytic gold particle from the initial VLS growth. Supplementary figure 4.b shows the I-V behavior of the samples with growth times of; 5 minutes (black line/ top panel), 10 minutes (blue line/ middle panel) and 30 minutes (red line/ bottom panel). Very little change in V_{OC} and *ff* is observed with increasing the diameter

from 90nm to 180nm; however the *ff* does decrease with a further increase in diameter to 215nm. The V_{OC} does not decrease with the increased surface area as would be expected, this is due to the resistance, as observed from impedance measurements, decreasing as the diameter increases, leading to a decrease in the resistance dependant voltage drop, allowing the two effects cancel each other out. The I_{SC} on the other hand increases dramatically from 1.5mA/cm^2 to over 5mA/cm^2, reaching a plateau for wire diameters of 150-180 nm and decreasing for the thickest wires. This trend in I_{SC} is shown by the red points in supplementary figure 4.c (the line is added as a guide to the eye). The blue plot shows the same data, but with the I_{SC} normalized to the nanowire surface area (again the line is added as a guide to the eye). From the I_{SC} and normalized I_{SC} plots it is apparent that for WZ GaP the optimum nanowire diameter, for PEC applications, is 150nm. This is an unexpected result, as our absorption measurements show an optimum absorption at 180nm (supplementary figure 4.c black plot). A decrease in absorption occurs at diameters greater than 180nm as reflection starts to occur at the top of the array[5]. Besides light absorption, bulk recombination also becomes a factor as the nanowire diameter increases past double the space charge width (as calculated in supplementary figure 1), resulting in a lower I_{SC} than expected for the larger nanowire diameters. The I_{SC} decreases further with diameter as the average refractive index is increased causing reflection to become an issue. A possible further reason for the lower I_{SC} from the thicker nanowires is the axial growth caused by the gold particle during shell growth. As the axial growth is not intentional during this growth phase stacking faults are incorporated, which could lead to recombination of charge carriers, and a lower than expected I_{SC}.

Supplementary note 2. Electrochemically Produced Oxide (EPO)

Due to the large surface area of the 2μm long 150nm wide nanowires, an oxide layer can help to passivate surface states[6–8], reducing surface recombination. A simple method for the production of an oxide layer is to apply a reducing potential to the GaP electrode while under illumination in an aqueous acid. The surface of the GaP will be reduced to gallium metal and phosphine. The gallium metal is then quickly oxidized by the aqueous acid to gallium oxide, thus forming an electrochemically produced oxide (EPO), similarly to the process observed on InP[7]. The formation of this EPO layer can be observed during electrochemical measurements, by the increase observed in the current. The chronopotentiometry measurement shown in supplementary figure 5.a demonstrates this clearly. In the first 150 seconds the current increases as the oxide layer is formed. Once the oxide layer is conformal over the surface of the nanowire the current stabilizes, and remains stable for the following 150 seconds. The experiment in supplementary figure 5.a is carried out under chopped illumination so that the dark current can also be observed. The fact that the dark current does not increase during the experiment shows that the current under illumination is purely due to the passivating effect of the oxide layer and not due to any surface charging, as that would also cause the dark current to increase. Supplementary Figure 5.b shows the I-V behavior of the nanowires after the production of the EPO layer, the I_{SC} and V_{OC} are both increased to 4.1mA/cm^2 and 0.75V (vs. RHE) respectively. The inset in this figure shows a TEM image of a section of a nanowire after the production of the EPO layer. The EPO layer is observed to be approximately 3nm in thickness, and is clearly not evident prior to the electrochemical treatment in figure 1.c in the main text. This EPO passivates surface states[6–8], decreasing surface recombination, leading to the observed increase in current.

In the presence of the EPO the I_{SC} is still limited to ~4mA, so a catalyst should still be implemented to promote charge transfer further.

Supplementary note 3. Catalyst deposition

Supplementary figure 7.a shows chronopotentiometry measurements performed on the nanowire samples after a single 180s deposition (black line), a single 60s deposition (red line) and after 3 consecutive 60s depositions (blue line). During the chronopotentiometry measurements, after a single 60s deposition (red line), an increase in current is observed over time. The increase is, however, not observed after 3 consecutive 60s depositions (blue line), and only a slight increase is observed for the 180s deposition (black line). It can be seen from supplementary figure 5 that the oxide layer increases slightly in thickness after 3 consecutive platinum depositions. When the platinum coverage is low, as is the case for 1x60s (and to a lesser extent for 1x180s), reactions will still occur on the nanowire surface without the aid of the catalyst. This, has in this case, lead to the oxide being reduced back to gallium metal, and exposing the GaP surface, allowing for the production of a thicker (passivating) oxide layer, which will act to reduce surface recombination and therefore increase current. Once the catalyst loading is high enough, as is the case for 3x60s, the catalyst particles are used preferentially for charge transfer from the semiconductor to the electrolyte, allowing for an increased reaction rate. The preferential use of the catalyst particles for charge transfer will also reduce the chance of oxide layer removal and surface reduction, leading to the reasonable stability observed in figure 3.c.

Supplementary References

1. Standing, a J., Assali, S., Haverkort, J. E. M. & Bakkers, E. P. a M. High yield transfer of ordered nanowire arrays into transparent flexible polymer films. *Nanotechnology* **23,** 495305 (2012).

2. Sim, U., Jeong, H.-Y., Yang, T.-Y. & Nam, K. T. Nanostructural dependence of hydrogen production in silicon photocathodes. *J. Mater. Chem. A* **1,** 5414–5422 (2013).

3. Maiolo, J. R., Atwater, H. a. & Lewis, N. S. Macroporous Silicon as a Model for Silicon Wire Array Solar Cells. *J. Phys. Chem. C* **112,** 6194–6201 (2008).

4. Osterloh, F. E. Inorganic nanostructures for photoelectrochemical and photocatalytic water splitting. *Chem. Soc. Rev.* **42,** 2294–320 (2013).

5. Kupec, J., Stoop, R. & Witzigmann, B. Light absorption and emission in nanowire array solar cells. *Opt. Express* **18,** 27589–27605 (2010).

6. Panda, J., Roy, A., Gemmi, M. & Husanu, E. Electronic Band Structure of Wurtzite GaP Nanowires via Resonance Raman Spectroscopy. *J. Am. Chem. Soc.* **135,** 1057–1064 (2013).

7. Munoz, a. G. *et al.* Photoelectrochemical Conditioning of MOVPE p-InP Films for Light-Induced Hydrogen Evolution: Chemical, Electronic and Optical Properties. *ECS J. Solid State Sci. Technol.* **2,** Q51–Q58 (2013).

8. Esposito, D. V, Levin, I., Moffat, T. P. & Talin, a A. H2 evolution at Si-based metal-insulator-semiconductor photoelectrodes enhanced by inversion channel charge collection and H spillover. *Nat. Mater.* **12,** 562–8 (2013).
